# Isolated Primary Rhinosporidiosis of the Parotid Duct: A Rare Presentation

**DOI:** 10.22038/ijorl.2020.43051.2408

**Published:** 2020-05

**Authors:** Swagatika Samal, Pradeep Pradhan, Chappity Preetam

**Affiliations:** 1 *Department of Pathology and Laboratory Medicine, All India Institute of Medical Sciences, *Pin-751019 *Bhubaneswar, Odisha, India.*; 2 *Department of Otorhinolaryngology, All India Institute of Medical Sciences, Bhubaneswar, Odisha, India, Pin-751019.*

**Keywords:** Parotid duct, Primary rhinosporidiosis, Surgical excision

## Abstract

**Introduction:**

The primary involvement of the parotid duct in rhinosporidiosis is very rare in clinical practice. Here, we present a case of rhinosporidiosis primarily involving the parotid duct, which was successfully excised through transparotid and transoral approaches.

**Case Report:**

A 51-year-old male presented with a painless progressive swelling over the left cheek for nine months. It was diagnosed as a parotid cyst or a mucous retention cyst based upon the radiological and cytological features. The cyst was completely excised with transparotid and transoral approaches, and the final diagnosis was confirmed to be rhinosporidiosis.

**Conclusion:**

Although the nose and the paranasal sinus are the common sites to be involved in rhinosporidiosis, the affection of the parotid duct is very unusual in clinical practice.

## Introduction

Rhinosporidiosis is a chronic granulomatous disease caused by Rhinosporidium seeberi (R. seeberi), frequently encountered in the southern zones of India and Sri Lanka (1). Nose and nasopharynx are the common sites affected by this disease, and the patients usually present with a painless mass with a history of epistaxis (2). It often presents as a polypoidal mass in the nasal cavity (3). In contrast, the primary involvement of the parotid duct is very rare in clinical practice, and very few cases have been reported in the literature worldwide. In the present case, we have reported an isolated parotid duct rhinosporidiosis presenting with cheek swelling, which was successfully managed by transparotid and transoral excision.

## Case Report

**Fig 1:**
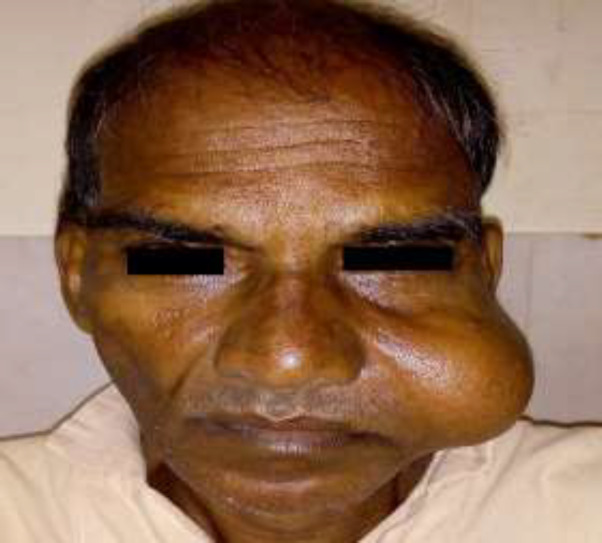
Photograph showing a globular **s**welling over the left cheek

A 51-year old male presented with a cheek swelling for nine months to the outpatient department. On local examination, the swelling was 4×5 cm in dimension (Fig. 1), which was non-tender and fluctuant on palpation. 

**Fig 2:**
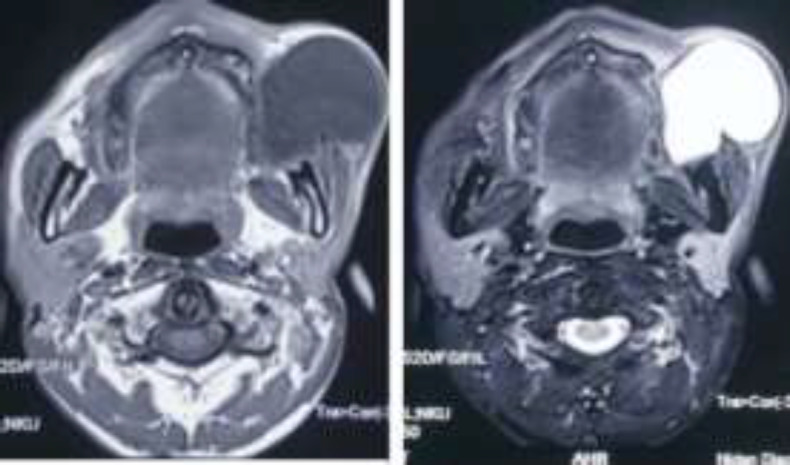
Contrast magnetic resonance imaging (T1 and T2 weighted) showing a thick-wall cystic swelling in the left buccal space over the temporalis muscle

Ultrasonography revealed a cystic lesion of 4.5×5.0 cm on the left side of the face in the subcutaneous plane. Routine clinical examination of ear, nose, and throat was found normal. Furthermore, Fine Needle Aspiration Cytology (FNAC) showed a benign cystic lesion, and contrast-enhanced magnetic resonance imaging demonstrated a non-enhanced, thick-walled cystic lesion (T1 and T2 weighted) presenting over the left temporalis muscle (Fig.2). 

**Fig 3:**
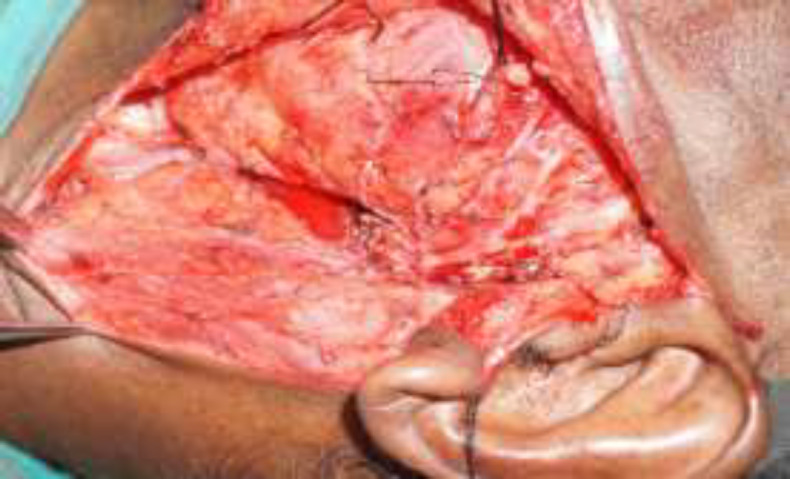
After superficial parotidectomy, the cyst was detected arising from the parotid duct (black arrow)

Moreover, the parotid gland was found normal, and it was provisionally diagnosed to be a ductocele arising from the left parotid duct or a mucous retention cyst 

based upon the radiological and pathological findings. After obtaining the informed written consent, the patient was planned for the excision of the cyst under general anesthesia. Modified Blair incision was given in the left preauricular area extending to the neck, two-finger breaths below the angle of the mandible. Superficial parotidectomy was performed after identifying the branches of the facial nerve (Fig.3). 

The parotid duct was identified at the anterior border of the master muscle, which was traced anteriorly towards the cyst. The cyst was dissected carefully from the intraoral attachments and later removed entirely. The histopathology of the excised specimen was confirmed to be rhinosporidiosis**.** The patient was on regular follow-up in the rhinology clinic for the past six months, where complete Ear, Nose, and Throat examination was carried out, including the diagnostic nasal endoscopy. He was found asymptomatic until six months in the follow-up period.

## Discussion

 Rhinosporidiosis is a chronic granulomatous disease affecting both humans and animals, caused by *R. seeberi*. It is frequently encountered in the southern parts of India and Sri Lanka. Nose and nasopharynx are the frequent sites to be affected by this disease (4). Isolated primary parotid duct rhinosporidiosis is very rare in clinical practice, and a few handfuls of cases have been reported in the literature worldwide. Although epistaxis and nasal obstruction are the common clinical presentations seen in the clinical practice, the patient can present with a cheek swelling, especially when it affects the parotid duct, as demonstrated in the present case. According to the previously conducted studies, only 6 cases have been documented in the literature across the world (5-10), and all the cases were diagnosed as parotid cyst before the final confirmation of diagnosis as rhinosporidiosis. 

**Fig 4:**
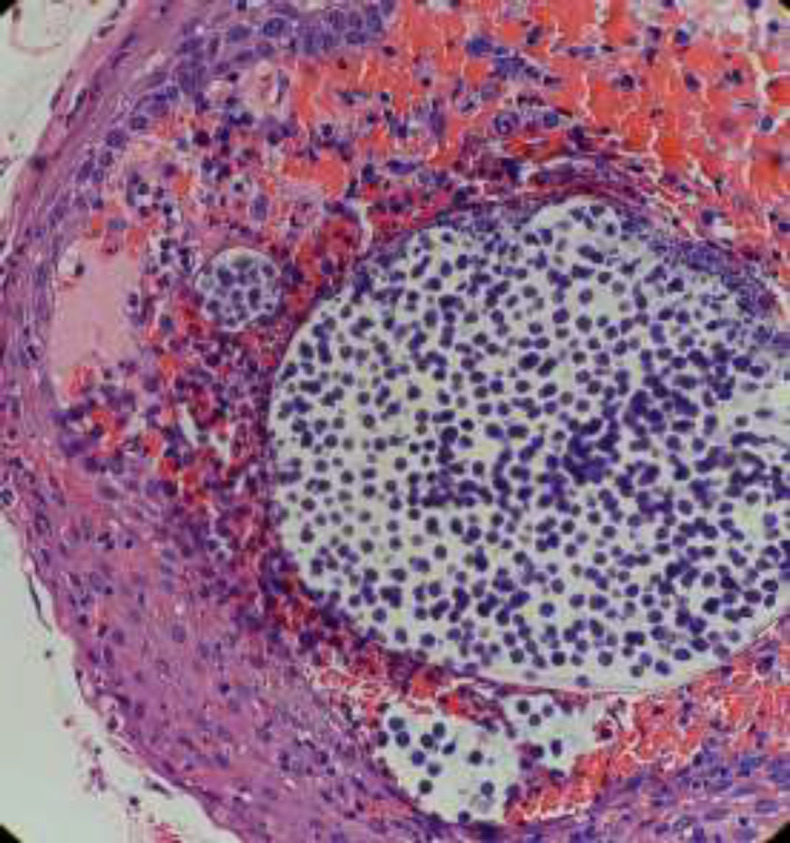
On histopathology, the lesion consists of globular cysts representing the sporangia containing daughter spores in different stages of development (Light microscopy, H&E, 50x)

 In the present case, the condition was also confused with the mucous retention cyst of the cheek or a ductocele as there was no definitive clinical and radiological feature of rhinosporidiosis. Diagnosis is always made by histopathological examination after the excision of the mass (11). On histopathology, the lesion has a distinctive morphology that consists of globular cysts representing the sporangia containing daughter spores in different stages of development (Fig. 4). 

 The organism is stained by periodic acid Schiff, Gomori methenamine silver, and mucicarmine (12). Although FNAC can be diagnostic in isolated parotid duct rhinosporidiosis as reported in the literature (13), it is not always confirmatory, as seen in the present case. The treatment is mostly by surgical excision with electrocautery of the base of the lesion. Very rarely, systemic dapsone can be offered in patients of systemic rhinosporidiosis where complete removal of the mass is a challenge. 

 In the present case, the complete excision of the cyst was successfully achieved using transparotid and transoral approaches. Although recurrence is common in rhinosporidiosis, which could be due to the spillage of the endoscopes during surgery, our patient was asymptomatic until six months of surgery without any recurrence of the disease. 

## Conclusion

 Although the nose and the paranasal sinus are the common sites to be involved in rhinosporidiosis, the affection of the parotid duct is very unusual in clinical practice. In the present case, we have documented a rare case of isolated primary parotid duct rhinosporidiosis, which was successfully excised using transparotid and transoral approaches.
